# Supplementary material for: Splice-Junction-Based Mapping of Alternative Isoforms in the Human Proteome
Source: Cell Rep. Author manuscript; Available in PMC 2020 Jan 15. (PMC6961840; doi:10.1016/j.celrep.2019.11.026)
Supplement: 2 [file NIHMS1546469-supplement-2.zip › DF1/PXD010154/Testis/Testis_32_CD99_EGEEEQGEVDMESHR.pdf]

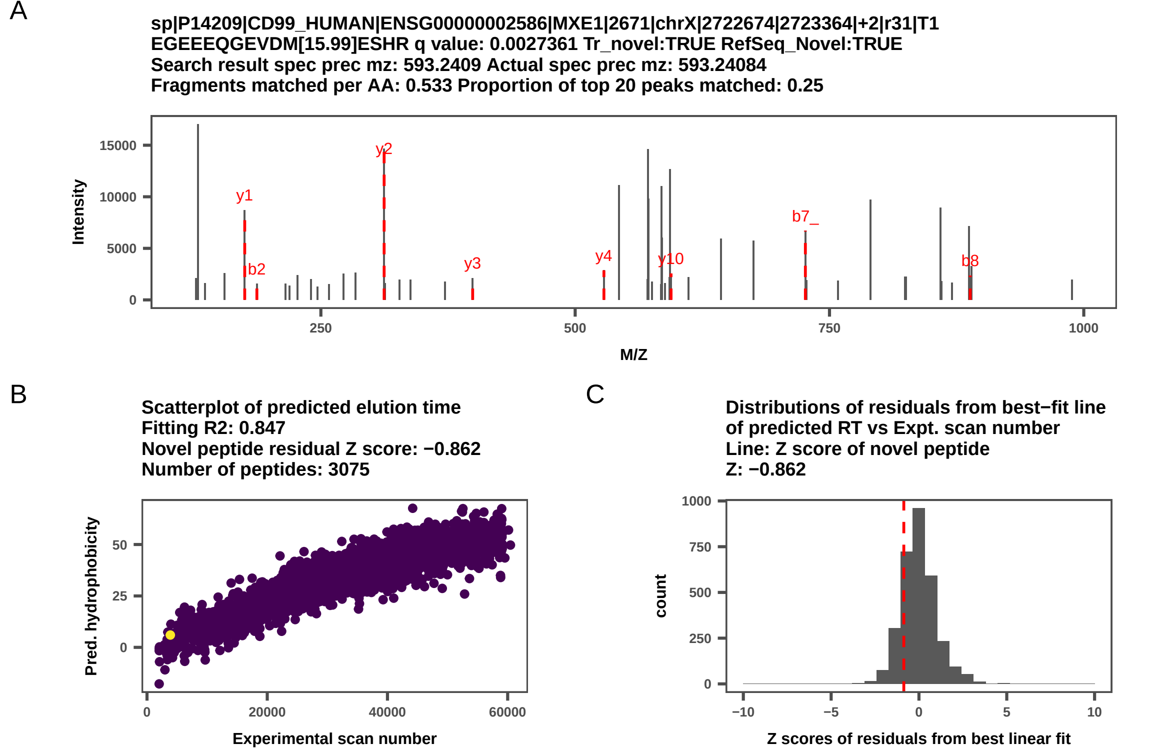
A
sp|P14209|CD99_HUMAN|ENSG00000002586|MXE1|2671|chrX|2722674|2723364|+2|r31|T1
EGEEEQGEVDM[15.99]ESHR q value: 0.0027361 Tr_novel:TRUE RefSeq_Novel:TRUE
Search result spec prec mz: 593.2409 Actual spec prec mz: 593.24084
Fragments matched per AA: 0.533 Proportion of top 20 peaks matched: 0.25
Intensity
M/Z
y1
b2
y2
y3
y4
y10
b7_
b8
B
Scatterplot of predicted elution time
Fitting R2: 0.847
Novel peptide residual Z score: −0.862
Number of peptides: 3075
Pred. hydrophobicity
Experimental scan number
C
Distributions of residuals from best−fit line
of predicted RT vs Expt. scan number
Line: Z score of novel peptide
Z: −0.862
count
Z scores of residuals from best linear fit
